# Beyond toolkits: sexual and reproductive health and rights literacy requires women‐centred structures, services and policies

**DOI:** 10.1002/jia2.25888

**Published:** 2022-03-07

**Authors:** Aditi Sharma, Agnes Ronan, Angelina Namiba, Ayu Oktariani, Laura Davies

**Affiliations:** ^1^ Independent Brighton & Hove UK; ^2^ PATA Cape Town South Africa; ^3^ 4M Mentor Mothers Network London UK; ^4^ IPPI Jakarta Indonesia

1

As the United Nations (UN) marks the 45th International Women's Day, why is it that women and girls living with HIV are still being denied their sexual and reproductive health and rights (SRHR)? We believe this is not only due to a lack of comprehensive services, but that low levels of health literacy among women and wider society are a significant factor.

Broadly, health literacy is the ability of individuals to obtain, understand and use information to take decisions and actions relating to their health. In 1998, WHO recognized that “health literacy means more than being able to read pamphlets and make appointments,” it also requires “the achievement of a level of knowledge, personal skills and confidence to take action to improve personal and community health by changing personal lifestyles and living conditions.” [1].

Health literacy must also include a focus on rights. In 2018, the Guttmacher report [2] emphasized that all individuals have the right to make decisions governing their bodies and that achieving sexual and reproductive health relies on realizing sexual and reproductive rights – which encompass a wide range of rights, from the freedom to define one's own sexuality to choosing whether, when and by what means to have a child.

Governments have made commitments to address and ensure SRHR [3] that are supported by global policies and guidelines, including for women living with HIV [4], community organizations and health workers [5, 6]. Yet, these commitments are only valuable if women themselves know, own and claim their rights. To this end, a multitude of SRHR literacy materials have been developed for women and adolescent girls living with HIV. Despite these tools, we are disheartened by the low levels of sexual and reproductive health literacy among women living with HIV, their communities and the health workers who support them. In Africa, overall health literacy varies greatly, from 8.5% in Niger to 63.9% in Namibia [7].

Information alone is not enough to enable women to make the best decisions for their own health. Before scarce funds are used to develop more SRHR literacy resources, we need to think afresh and consider questions like: Will a new resource reach the woman who needs it? Will it connect with her life? Will she have the support needed to use and benefit from the information? Investment in the following five areas could transform health literacy and, as a result, outcomes for women living with HIV.

As the definition of health literacy recognizes, women need the knowledge, skills and confidence to decide what is best for them. But this is not simple. There are many ways that our cultures, traditions and laws prevent women from having the power to make their own decisions. We need to support women's organizations and their efforts to shift power towards women and change the attitudes and norms that deny women equal rights. Interventions should promote an individual woman's agency and ensure she is meaningfully involved in decisions about her body and health. The 4M Network of Mentor Mothers bring their toolkits to life through training, peer mentoring and providing spaces for women to lead, speak and facilitate [8]. Recent WHO guidelines to promote self‐care recognize women as active agents in managing their health, giving them greater control over their choices and decisions [9].

SRHR literacy initiatives must reflect the ways in which women learn, where they learn best and who they trust. Diverse communities of women living with HIV should take the lead – they are the trusted experts [10]. We need more political and financial backing for structures and initiatives that are led by, and support women living with HIV to know and claim their rights [11]. For example, funding should go to support women to create safe spaces where they are comfortable to meet and share their personal experiences. This includes paying for the things that make it easier for women to gather together, such as providing menstrual products, childcare, transport costs, coffee or snacks. These are interventions that may improve SRHR outcomes more than new Information, Education and Communication materials.

SRHR literacy must reach all women in their diversity and be adapted to reflect the changing needs of women living with HIV through their life course, from adolescent‐specific information to services to prevent vertical transmission, and menopause support. Marginalized women, such as refugees, migrant women, women who use drugs and sex workers, who are often excluded from health literacy efforts must be prioritized. It is also important to reach those who influence women's decision making. We need SRHR literacy for adolescent boys and men with a focus on power, social and gender norms. We must ensure health workers have the knowledge and skills to provide stigma‐free and non‐discriminatory services. And policy makers need to understand the importance of ensuring SRHR for all, so that issues such as abortion, sexual and gender identity and comprehensive sexuality education are shared priorities, rather than being contested at high‐level policy meetings, such as at the UN [12].

Although communities are in the best position to lead SRHR literacy efforts, it should not just be left to the communities to champion or implement them. Governments need to work across ministries and sectors to build accountable health systems that enable all women living with HIV to realize their SRHR. We need investment in community feedback and monitoring mechanisms, such as the READY score cards [13], which guide actions to improve the quality of support that health workers provide for young people to make informed decisions.

In far too many countries, laws and policies undermine SRHR goals – such as those that require young girls or married women to get permission from others to access family planning services. Women living with HIV, including those from marginalized communities, face high levels of discrimination and human rights violations – from denial of antenatal care to coerced sterilization [14]. SRHR is not just about providing medicines and contraceptives but about re‐shaping social norms, policies and legal environments to reduce violence, stigma and gender inequities.

To mark International Women's Day, we urge donors, UN agencies and non‐governmental organizations to commit to SRHR literacy initiatives that improve knowledge and understanding, as well as address power imbalances and inequities so that women living with HIV can claim and realize their SRHR. This requires understanding the diverse contexts and lives of women living with HIV and investing beyond the information itself into the structures, services and policy environment.

## COMPETING INTERESTS

None of the authors have any competing interests.

## AUTHORS’ CONTRIBUTIONS

All authors contributed to conceptualizing the paper, reviewed drafts and approved the final version. AS led on writing the paper and LD helped with editing.

AS is an independent consultant and activist working with community organizations and UN agencies to advance health, human rights and social justice. AR is Head of Programmes at Paediatric Adolescent Treatment Africa (PATA) that aims to mobilize, strengthen and build resilience in a network of frontline health providers, facilities and communities on the frontline of paediatric and adolescent HIV service delivery in sub‐Saharan Africa. AN is a Co‐Director of 4M Mentor Mothers Network, a peer‐run organization of women living with HIV ensuring sexual and reproductive rights during pregnancy and beyond, for life. AO is the National Coordinator of Indonesia Positive Women Network (IPPI) working on advocacy and empowerment of women living with HIV. LD is an independent consultant who specializes in writing, designing and developing accessible tools and supporting communities of people living with HIV.

## FUNDING

None.

## DISCLAIMER

None.
